# Organ-on-a-disc: A platform technology for the centrifugal generation and culture of microphysiological 3D cell constructs amenable for automation and parallelization

**DOI:** 10.1063/5.0019766

**Published:** 2020-10-01

**Authors:** Stefan Schneider, Florian Erdemann, Oliver Schneider, Thomas Hutschalik, Peter Loskill

**Affiliations:** 1 Fraunhofer Institute for Interfacial Engineering and Biotechnology IGB, Nobelstrasse 12, 70569 Stuttgart, Germany; 2 Department of Women's Health, Research Institute for Women's Health, Faculty of Medicine, Eberhard Karls University Tübingen, Calwerstrasse 7, 72076 Tübingen, Germany

## Abstract

Organ-on-a-chip (OoC) systems have evolved to a promising alternative to animal testing and traditional cell assays in drug development and enable personalization for precision medicine. So far, most OoCs do not fully exploit the potential of microfluidic systems regarding parallelization and automation. To date, many OoCs still consist of individual units, integrating only one single tissue per chip, and rely on manual, error-prone handling. However, with limited parallelization and automation, OoCs remain a low-throughput technology, preventing their widespread application in industry. To advance the concept of microphysiological systems and to overcome the limitations of current OoCs, we developed the Organ-on-a-disc (Organ-Disc) technology. Driven only by rotation, Organ-Discs enable the parallelized generation and culture of multiple 3D cell constructs per disc. We fabricated polydimethylsiloxane-free Organ-Discs using thermoplastic materials and scalable fabrication techniques. Utilizing precisely controllable centrifugal forces, cells were loaded simultaneously into 20 tissue chambers, where they formed uniform cell pellets. Subsequently, the cells compacted into dense 3D cell constructs and were cultured under vasculature-like perfusion through pump- and tubing-free, centrifugal pumping, solely requiring a low-speed rotation (<1 g) of the Organ-Disc. Here, we provide a proof-of-concept of the Organ-Disc technology, showing the parallelized generation of tissue-like cell constructs and demonstrating the controlled centrifugal perfusion. Furthermore, Organ-Discs enable versatile tissue engineering, generating cell constructs with a customizable shape and a layered multi-cell type structure. Overall, the Organ-Disc provides a user-friendly platform technology for the parallelization and automation of microphysiological systems, bringing this technology one-step closer to high-throughput applications in industry.

## INTRODUCTION

Over the past few years, microfluidic Organ-on-a-chip (OoC) technology, replicating key aspects of human physiology,^1^ has become a promising toolbox for preclinical research, which is starting to get implemented by a growing number of pharmaceutical companies for drug development.^2^ OoCs, a type of microphysiological system, culture human microtissues with physiological structures under vascular perfusion in a tissue-specific, precisely controlled microenvironment.^3^ This technology can be personalized by combining OoCs with patient-derived cells^4,5^ or cells derived from patient-specific, induced pluripotent stem cells,^6^ providing a powerful tool for precision medicine. However, OoCs have not yet been applied for personalized medicine, as, e.g., limited handling automation and low-throughput character of the available systems represent major technical obstacles.^7^ Compared to current high-throughput screening performed by pharma and biotech companies, acquiring more than 100 000 data points per day,^8^ OoCs offer lower throughput by several orders of magnitude. This can be attributed to several reasons: many OoCs are fabricated using polydimethylsiloxane (PDMS), a material limiting scale-up of chip fabrication^9^ and known for its tendency to absorb small hydrophobic molecules,^10^ which can limit their usability for drug testing.^11^ Further limiting factors of current OoC systems, independent of the applied chip material, are low degrees of parallelization and automation.^9^

Therefore, several attempts were made to create OoCs with higher throughput capabilities: many of those approaches focus on the increase in the replicate number per chip and maintaining compatibility to standard lab equipment by tailoring those systems to the standardized footprint of a microtiter plate.^12,13^ Other concepts try to facilitate handling of OoCs by, e.g., on-chip peristaltic pumping^14,15^ or gravity-driven perfusion by periodical tilting of the chip.^16^ Additionally, fluid coupling between individual OoCs using liquid handling technology instead of manual pipetting for fluidic interconnections was just recently reported.^17^

Concepts from microfluidic cell culture systems with high degrees of parallelization^18^ and automation^19^ show the general high-throughput potential of microfluidic systems, achievable through a highly branched and complex channel network. However, those multiplexed systems lack physiologic relevance^9^ and often require multiple tubing connections, which are disadvantageous regarding, e.g., handling, leakage, and increase in the dead-volume.^20^

A tubing-free alternative for microfluidic high-throughput applications is provided by the Lab-on-a-disc (LoD) technology. LoD systems are capable of performing, e.g., highly parallelized immunoassays^21,22^ or digital polymerase chain reactions^23^ only based on rotation of a disc-shaped microfluidic module. So far, LoDs have mainly drawn attention for screening applications.^24,25^ However, researchers presented centrifugal systems, establishing unit operations that are in principle also required for OoC systems, such as cell loading or medium perfusion: centrifugal cell loading has been reported for single-cell assays,^26,27^ to monitor cell proliferation^28^ or to generate multi-cell type spheroids.^29^ Centrifugal pumping^30^ has furthermore been used for medium exchange of a culture chamber with adherent cells^31^ or for the cultivation of *Caenorhabditis elegans*.^32^ By inserting classical OoC modules in conventional centrifuges, we could recently demonstrate the general compatibility of centrifugal microfluidics and OoC technology and applied centrifugal cell loading to distribute cells into several tissue chambers, supplied by a syringe pump-driven medium flow.^33^

Here, we present a novel, entirely rotation-based Organ-on-a-disc system (Organ-Disc) as enabling platform technology for the pump-free generation and culture of human 3D cell constructs. A straightforward three-step process was established to generate and culture 3D cell constructs in the Organ-Disc: first, a cell suspension is pipetted into the inlet region of the Organ-Disc's tissue channels. Subsequent rotation of the Organ-Disc transports the introduced cells via centrifugal forces into the tissue chambers, located in the peripheral disc region, where they form dense cell pellets. In the third step, the Organ-Disc rotates at low speed with centrifugal forces well below 1 g to provide a centrifugally driven media flow. This creates a vasculature-like perfusion of cell culture medium through the media channels that are separated from the tissue chambers by a porous membrane. We characterized the Organ-Disc's centrifugal perfusion by theoretical considerations as well as experimental flow rate measurements. Our novel approach distinguishes itself through its easy adaptability, as all process steps can be tailored to a wide range of specific cell types. We demonstrate this by providing a proof-of-concept of the Organ-Disc technology's ability to generate and culture dense 3D cell constructs, incorporating patient-derived fibroblasts (FBs) and adipose tissue-derived stem cells (ASCs). Additionally, we demonstrate that the Organ-Disc allows for customizing the shape of 3D cell constructs and the targeted patterning of multi-cell types into a predefined cell layer structure.

## RESULTS AND DISCUSSION

### The rotation-based Organ-Disc technology

**FIG. 1.**
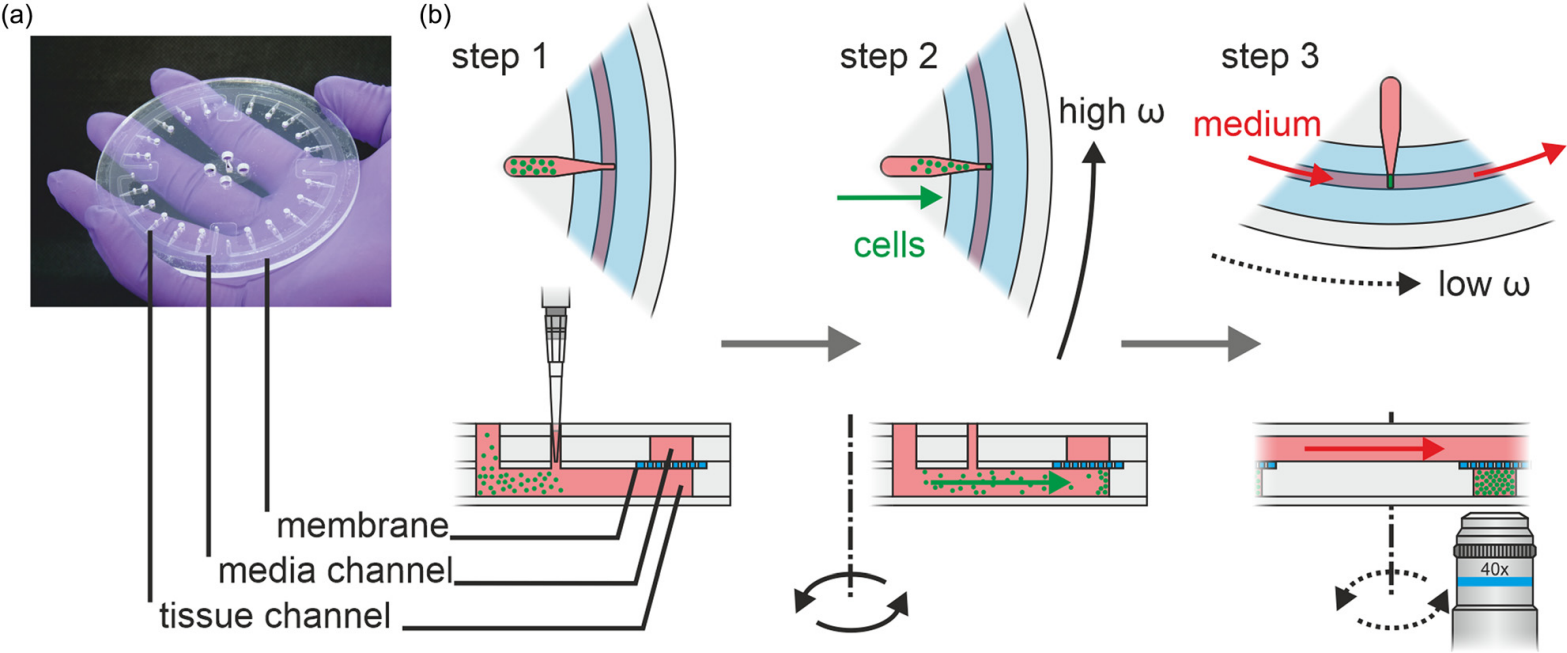
The Organ-Disc technology: (a) photo of an Organ-Disc with a 10 cm outer diameter. Each disc has four independent systems for parallelized 3D cell construct generation and culture. The independent systems consist each of five tissue chambers and a media channel. Organ-Discs are manufactured using thermoplastic materials and rapid prototyping techniques. (b) First step of the Organ-Disc application: a cell suspension is flushed into the tissue channels by a micropipette. Second step: rotation of the Organ-Disc transports cells from the inlet section into tissue chambers, forming dense cell pellets. Third step: slow disc rotation (<1 g) generates a controlled media perfusion. The porous membrane allows for diffusive transport and eliminates shear stress on the 3D cell constructs. The Organ-Disc enables automated readout technologies during culture, as all tissue chambers are symmetrically arranged on the disc and permanently optically accessible with an optical distance of only 175 *μ*m.

Organ-Discs are based on multi-layered, microfluidic modules. Each disc has a diameter of 10 cm and consists of three main components: a tissue layer, a media layer, and a porous membrane sandwiched in between. Each Organ-Disc features four independent systems comprising each five tissue chambers, connected in-line by a shared media channel [Fig. 1(a)].

The tissue layer features in total 20 identical tissue channels pointing radially outward. The channels are accessible through two loading ports by a micropipette. Each channel ends with a microcompartment, the tissue chamber, located in the peripheral disc region. Here, we have implemented two designs for different types of tissues: Rectangular tissue chambers provide a structural environment for single-cell type or layered multi-cell type constructs. The formation of an anisotropic fiber-like 3D cell construct with adhesion spots on both ends is enabled via dogbone-shaped chambers, adapted from previously introduced heart-on-a-chip systems.^33,34^ Both tissue chambers are 175 *μ*m high each and have a footprint of approximately 0.3 cm^2^ for the dogbone shape and approximately 0.5 cm^2^ for the rectangular design.

The media layer features four independent channels (1.4 mm wide and 75 *μ*m high) for the transport of cell culture medium. Each media channel supplies five tissue chambers in-line. A thin isoporous membrane (pore diameter 3 *μ*m) separates the media channels from the tissue chambers. As previously shown, the porous membrane allows for a diffusive exchange of nutrients to the microchambers, transported in the media channel, and, at the same time acts, as a barrier, protecting cells from excessive shear stress.^33,35^

Organ-Discs have been fabricated solely based on thermoplastic materials instead of PDMS. A well-known consequence of using microfluidic systems based on thermoplastics is, e.g., reduced gas permeability and its impact on oxygen concentrations in microfluidic systems.^36^ However, using thermoplastic materials enables the application of scalable, rapid prototyping techniques. Here, all layers, except for the media layer, were structured using a CO_2_ laser, resulting in precise structures with minimal lateral dimensions of approximately 150–200 *μ*m. The media layer, having a less complex design and larger lateral dimensions as the tissue layer, allowed for a less sophisticated structuring method. Instead of laser cutting, we structured the media layer with a knife plotter. At the same time, knife plotting allowed for smoother channel edges compared to laser structuring, minimizing trapping of air bubbles during initial flushing. All structuring steps for one disc took in total less than 1 h. The individual, thermoplastic layers of the Organ-Disc were bonded in approximately 2 h using solvent vapor bonding.

Using Organ-Discs, 3D cell constructs are generated and cultured in three simple steps: first, cells are pipetted into the inlet region of the tissue channels [Fig. 1(b)]. Then, dense cell pellets are generated in all tissue chambers simultaneously by precisely controllable centrifugal forces. Finally, a continuous perfusion is provided by centrifugal pumping, relying on a slow disc rotation with centrifugal forces below 1 g. A rotation-based system involves more effort in imaging during rotation compared to a stationary chip. However, the symmetrical arrangement of tissue chambers on the Organ-Disc and a minimal optical distance of approximately 175 *μ*m to the cells enable, in principle, an automated monitoring during centrifugal culture. By adding short phases of stepwise rotation of the disc, a stationary microscope lens would be sufficient for imaging all tissue chambers one after each other.

### Pump- and tubing-free centrifugal perfusion

**FIG. 2.**
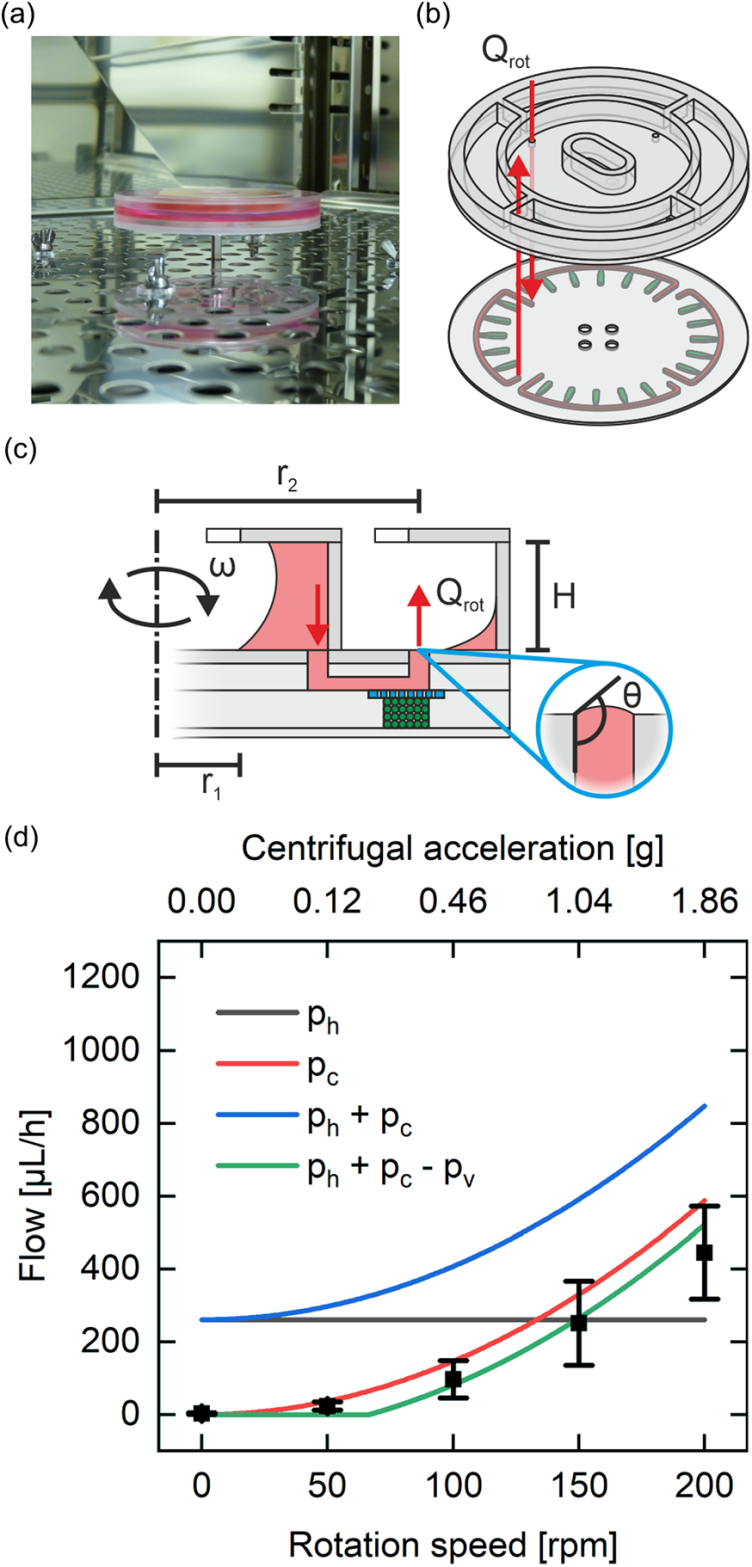
Concept and characterization of centrifugal perfusion: (a) photo of a rotating Organ-Disc with the reservoir on the perfusion spinner inside an incubator. (b) Exploded view of the reservoir connection to the Organ-Disc. The reservoir's ports are connected to the inlet and outlets of the media layer, providing fresh medium from the central compartment and collecting the effluent of each system separately in outer compartments. The reservoir is shown without its lid. (c) Via rotation, medium is pumped through the media channels by centrifugal pressure. Medium inside the compartments is pushed radially outward during rotation, resulting in an additional, constant hydrostatic pressure difference. The exit port to the effluent compartments acts as a capillary burst valve. (d) Comparison between measured (squares, N ≥ 8, error bars represent the standard deviation) and calculated flow rates (lines) during centrifugal perfusion. Flow rates were calculated considering hydrostatic only (black), centrifugal only (red), and hydrostatic and centrifugal pressure differences (blue), as well as hydrostatic and centrifugal pressure differences reduced by an approximated, capillary pinning effect (green).

The Organ-Disc technology allows for a continuous perfusion without the need for external pumps or tubing connections to the disc. Instead, we applied centrifugal pumping,^30^ solely requiring rotation of the microfluidic system. For centrifugal media perfusion, we built a perfusion spinner, allowing for rotation of the Organ-Disc and its reservoir at low speed inside an incubator [Fig. 2(a)]. The Organ-Disc's reservoir features in total five compartments that are accessible from the top and store liquid during centrifugal pumping. Here, a single compartment in the center of the reservoir, connected to the inlets of each media channel, stores and supplies fresh cell culture medium to each system. However, it should be noted that independent culture conditions are, in principle, easily achievable by further segmentation of this inner compartment. The other four compartments, located at the periphery of the reservoir, are connected to the outlets of the media channels and collect the effluent of each system [Fig. 2(b)].

Centrifugal perfusion has to meet several requirements in order to be applicable for OoC systems. First, the centrifugal forces, inevitably present during rotation and permanently acting on the cells, must not immensely exceed 1 g, otherwise resulting in unintended hypergravity conditions. However, those centrifugal forces need to be sufficient to generate flow rates at similar levels compared to other OoC systems. Therefore, we focused on suitable parameters for the centrifugal perfusion of the Organ-Disc by theoretical calculations as well as flow measurements.

Rotation of the Organ-Disc leads to a pressure on the liquid (density *ρ*) inside the media channels and the reservoir, which depends on their radial position [Fig. 2(c)]. Therefore, rotation with an angular velocity *ω* leads to a centrifugal pressure difference

$$\Delta p_{c}=\frac{1}{2}\rho \omega^{2}\left(r_{2}^{2}-r_{1}^{2}\right),$$ (1)

between the inner radius (*r*_1_) and the outer radius (*r*_2_) of the liquid volume, which is proportional to *ω*^2^.^24^

Before each centrifugal perfusion, the inner compartment of the reservoir is filled to the brim, while the outer compartments are initially empty. Due to the difference in fluid level *H*, acting perpendicular to the rotation of the disc, a hydrostatic pressure difference

$$\Delta p_{h}=\rho gH,$$ (2)

is present during centrifugal media perfusion. All fluids in the reservoir are pushed radially outward during rotation of the Organ-Disc. Therefore, the fluid level above the inlet remains maximum, as fluid is constantly present up to the brim. However, the fluid level above the outlet remains zero as the effluent gets pushed past the port, respectively [Fig. 2(c)]. Hence, for the following calculations, we assume a constant hydrostatic pressure if the disc and its reservoir are spinning.

The volumetric flow, generated by Δ*p_c_* and Δ*p_h_*, through the disc's rectangular media channel (height *h*, width *w*, whereby *h* ≪ *w*, and length *L*)^37^ can now be calculated for fluid with dynamic viscosity *η* as follows:

$$Q=\frac{h^{3}w\left(\Delta p_{c}+\Delta p_{h}\right)}{12\;\eta L}\left[1-0.630\frac{h}{w}\right].$$ (3)

Here, we calculated Δ*p_c_* using the middle of the inner compartment for *r*_1_, as compromise between full and empty inner compartments and the media channel outlet for *r*_2_, assuming a local separation between medium in the channel and the collected effluent. Further, we calculated Δ*p_h_* for the 8 mm fluid level of water in the inner compartment at 37 °C. This leads to a hydrostatic pressure difference of approximately 78 Pa.

In order to verify our theoretical assessment, we measured the actual flow rate generated by centrifugal pumping by weighing the effluent. The experimentally measured flow rates reached from almost undetectable flow rates of 3 *μ*l/h for 0 rpm up to 445 *μ*l/h at the highest tested velocity of 200 rpm [Fig. 2(d)]. Already at 100 rpm, a flow rate of 97 *μ*l/h was achievable, comparable—or even above—conventionally used values.^33,35,38^ Using a rotation speed of 100 rpm leads to a centrifugal force of 0.46 g, acting on the cells. Considering both centrifugal acceleration and standard gravity and combining them by vector addition, the cells sense only a minimal evaluated gravitation of 1.1 g at 100 rpm.

As expected, our measurements reveal a superlinear correlation between the flow rate and rotation speed. However, the measured flow rates are much lower than the calculated flow rates, generated by centrifugal and hydrostatic pressure.

Several aspects could potentially reduce the volume flow. First, a discrepancy between input and actual output rotation speed might be present due to the open-loop controlled perfusion spinner. In addition, evaporation might reduce the amount of liquid, collected over time in the outer compartments of the reservoir. Further, there is a short but inevitable phase between stopping the rotation and weighing the effluent that is undefined flow condition. However, the most likely explanation in our opinion is capillary pinning,^30^ acting as an additional resistance, reducing the actual pressure difference between the inlet and the outlet.

Capillary pinning can occur at an abruptly diverging channel geometry. This effect is often used for capillary burst valves that prevent fluids from further advancing unless a certain burst pressure is exceeded.^30^ In the case of the Organ-Disc, it is plausible that the meniscus of the liquid traveling up the port to the outer reservoir is pinned at the top edge, whereby the ports to the outer compartments would create a capillary burst valve. Considering that the effluent is pushed radially outward during rotation, capillary pinning could be a permanent effect as long as no liquid is accumulated above the exit port, resulting in a permanent backpressure [Fig. 2(c)].

Cho *et al.* estimate the maximum burst pressure *p_v_* of liquid, pinned inside a circular capillary using

$$p_{v}=-4\sigma \frac{\text{cos}\theta_{\text{max}}}{d},$$ (4)

with *σ* being the surface tension of the liquid, *d* the inner diameter of the capillary, and *θ_max_* the maximum contact angle that the liquid can build up before spreading in the diverging section.^39^ According to the author's equation and their assumption of *θ_max_* = 180°, a maximum burst pressure of up to 140 Pa for our circular reservoir ports with a diameter of 2 mm is possible. Consequently, no flow should be observable up to approximately 120 rpm when the total pressure difference exceeds the burst pressure. However, this was not observed in the experiments and could be due to the assumption of a maximum contact angle of *θ_max_* = 180°.

If the liquid wets the surfaces perpendicular to the capillary valve opening, the burst pressure is reduced.^30^ Since the reservoir is made out of polymethyl methacrylate (PMMA), known as a hydrophilic polymer,^40^ using the equation from the study by Cho *et al.* with *θ_max_* = 180° might lead to an overestimation of *p_v_* in our system. In addition, multiple studies concluded that surface roughness and rounded edges at the diverging geometry reduce the capillary pinning effect.^30,39,41^ As the ports are fabricated in a rapid-prototyping manner by laser cutting, this might result in further reduction of the burst pressure in our system.

We, hence, fitted our measured flow rates with a variable burst pressure assuming permanent capillary pinning. We used

$$Q=\left\{\begin{array}{l} 0\\ B\left(\omega^{2}-\omega_{0}^{2}\right) \end{array}\right.\begin{array}{c} \text{for}\;\omega <\omega_{0}\\ \text{for}\;\omega \geq \omega_{0}, \end{array}$$ (5)

as a function for the approximation of our measurements, with

$$\omega_{0}^{2}=\frac{p_{v}-p_{h}}{\frac{1}{2}\rho \left(r_{2}^{2}-r_{1}^{2}\right)},$$ (6)

being the angular velocity at which the capillary valve bursts and

$$B=\frac{h^{3}w}{12\;\eta L}\left[1-0.630\frac{h}{w}\right]\left[\frac{1}{2}\rho \left(r_{2}^{2}-r_{1}^{2}\right)\right],$$ (7)

a parameter, only depending on channel dimensions and material properties. The error-weighted approximation indicates a burst pressure of 97 ± 17 Pa, slightly higher than the hydrostatic pressure. Comparing our measurements with this fit of a flow rate induced by centrifugal as well as hydrostatic pressure and reduced by permanent capillary pinning gives a better matching with a coefficient of determination *R*^2^ = 0.7 [Fig. 2(d)].

### Parallelized centrifugal cell loading and versatile 3D cell construct culture

For cell loading, only a single pipetting step is necessary in which a cell suspension is transferred into each tissue channel through the inlet ports. The actual 3D tissue generation is accomplished by subsequent centrifugal cell loading in all cell channels simultaneously: the Organ-Disc rotates and the occurring centrifugal forces transport the cells into the tissue chamber at the outer end of each tissue channel. By filling the outer tissue chambers, dense cell pellets are created in shapes predefined by the chamber geometry.

This is different from conventional OoCs, relying on the injection of a cell suspension by pressure-driven flow generated, e.g., by pumps or micropipettes.^5,6,42^ In general, those OoCs load cells based on filtration as cells are pumped into a microchamber and retained by a porous material. In such a process, the excess liquid needs to pass the membrane pores in order to maintain a fluid flow for further cell transport into the chamber. As the accumulating cells will clog the pores of the membrane, gradually increasing pressure gradients will be needed for further cell accumulation. Therefore, such a filtration-based process depends on the current filling state of the tissue chamber and might require the application of large, potentially cell damaging pressures for a complete filling of a tissue chamber.

In contrast to that, the Organ-Disc cell loading is solely based on sedimentation through defined centrifugal forces. On the one hand, this allows for the integration of all injected cells into the cell construct, as they are actively transported into the tissue chamber without increasing resistance. This allows us to generate a dense pellet with only 20 000–40 000 cells, depending on the tissue chamber geometry and cell type. Thereby, more than 20 3D cell constructs can be generated with less than one million cells, making the Organ-Disc an efficient system in cell demand. On the other hand, the applied centrifugal forces acting on the cells during centrifugal loading are precisely controlled by the rotation speed of the Organ-Disc. Different from filtration-based OoCs, the Organ-Disc allows thereby for a gentle cell injection without exerting pressure gradients or applying shear-forces on the cells.

**FIG. 3.**
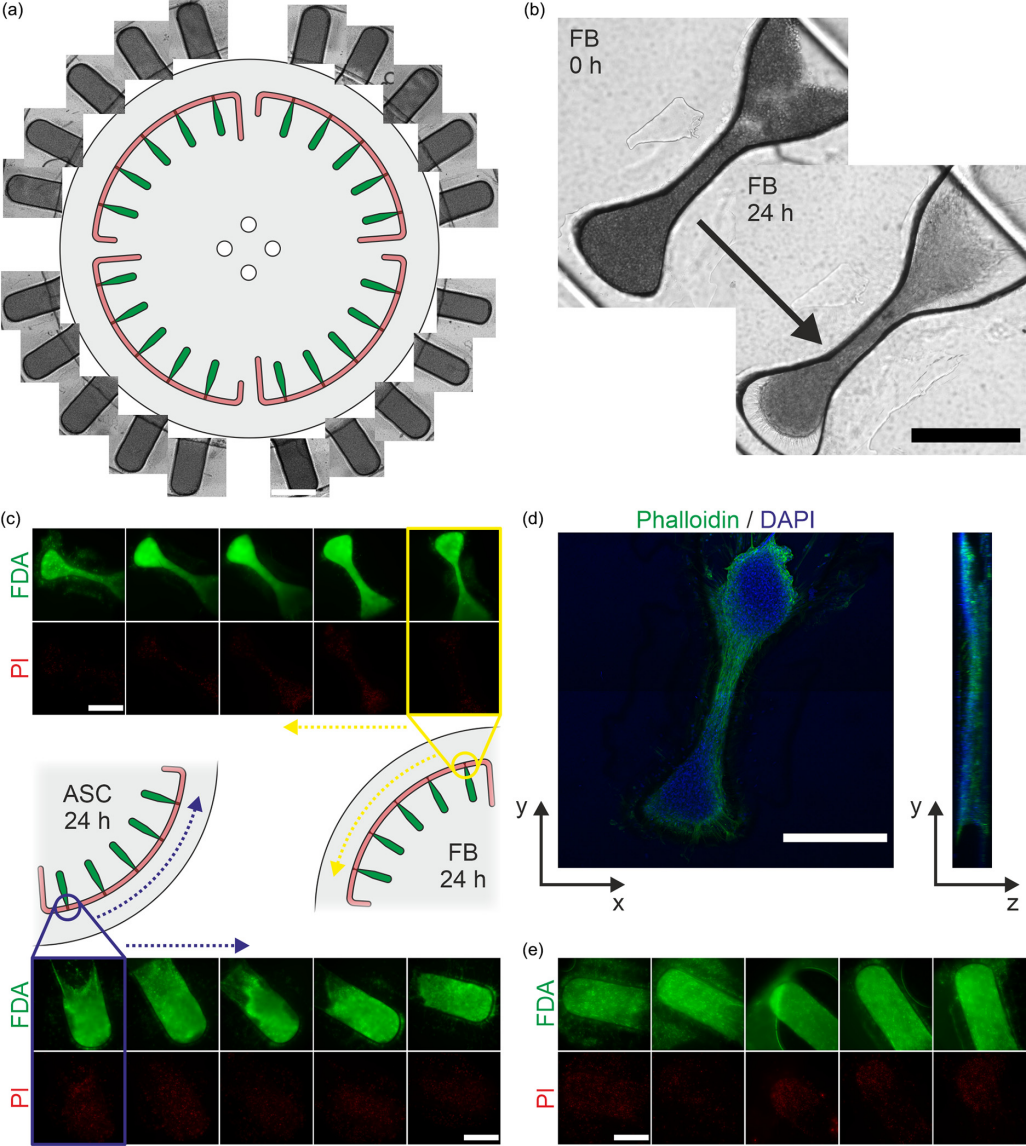
Centrifugal 3D cell construct generation and culture: (a) centrifugal cell loading with ASCs. All tissue chambers of an Organ-Disc were evenly filled with ASCs after 5 min of spinning at 1000 rpm. Shown are bright field images of all the 20 tissue chambers of one disc. (b) FBs compacting into a tissue-like, 3D cell construct. After 24 h, the injected, individual cells attached to other cells in their vicinity, forming a connected multi-cellular structure. Shown are bright field images of the same tissue chamber immediately after loading and after 24 h of subsequent culture (100 rpm, 0.46 g). (c) Live/dead (FDA/PI) staining of FB and ASC cell constructs after one day of centrifugal culture (100 rpm, 0.46 g). Successful generation of viable cell constructs in dogbone-shaped and rectangular tissue chambers. Shown are all the five FB or ASC tissue chambers of one system. FBs and ASCs were cultured on separate Organ-Discs. Shown are maximum intensity projections after approximately 24 h. (d) Actin and nuclei (phalloidin/DAPI) staining of a FB cell construct cultured for 24 h (100 rpm, 0.46 g). Shown are maximum intensity projections in z- and x-directions. The 3D structure is predefined by the dogbone-shaped tissue chamber, enabling an anisotropic fiber formation with round anchor points on both sides. (e) Live/dead staining of ASCs cultured for three days by centrifugal perfusion (100 rpm, 0.46 g). All the five 3D cell constructs of one in-line supplied system were overall FDA-positive. An air bubble is observable in the media channel, entrapped during the staining procedure, however not affecting cell viability. Shown are maximum intensity projections after approximately 72 h. Scale bars: 500 *μ*m.

Using centrifugal cell loading, we successfully generated cell constructs with two different geometries: we loaded FBs into dogbone-shaped compartments and ASCs into rectangular tissue chambers. Therefore, the structural environment and support, provided by the tissue chamber, can be flexibly tailored to a specific application. In general, the Organ-Disc loading process is easily adaptable to a specific cell type, requiring an optimized cell number for complete filling of a tissue chamber or higher forces for building a dense cell pellet. For the cell types used in this work, 5 min of spinning at 1000 rpm was sufficient to transport the cells into the tissue chambers and to form 20 uniform pellets in one step [Fig. 3(a)]. The centrifugal acceleration acting on the cell pellets inside the microchamber during loading is approximately 46 g. This allowed us to fill entire discs at once with centrifugal forces below 100 g, a commonly used acceleration in cell culture for, e.g., passaging cells.^43^

After 24 h of centrifugally perfused culture (rotation at 100 rpm or 0.46 g), the formation of tissue-like, 3D cell constructs could be observed using both cell types and tissue chamber designs. The cells changed from their spherical shape into elongated shapes attached to other cells in their vicinity forming a connected multi-cellular structure [Fig. 3(b)]. Cell viability was evaluated after 24 h of culture via live/dead staining with FDA and PI. It was possible to generate systems on the Organ-Disc only containing viable 3D cell constructs for both cell types. All the five tissue chambers, connected in-line to each other in one system, contained minimal amounts of dead cells in comparison to the overall viable and FDA-positive cells [Fig. 3(c)].

We occasionally observed cells migrating in between the membrane and the tissue layer. This is due to local delamination of the membrane from the tissue layer material. Therefore, cells can enter the created gaps. While this indicates room for improvement of the adapted bonding process for membrane integration, we have not seen a negative impact on the cell viability. The cells in those gaps have uncompromised access to the perfused cell culture medium, as they are directly underneath the porous membrane. Therefore, neither the cells in those gaps nor the associated cells inside the tissue chambers showed reduced viability.

Already after 24 h of centrifugal culture, the cells adapted to the predefined structure of our tissue chambers. In the case of the dogbone-shaped chamber, the microchamber was completely filled with FBs aggregated to a single 3D cell construct with an anisotropic fiber-like structure [Fig. 3(d)]. The resulting thickness of the 3D construct, predefined by the height of the tissue chamber, was approximately 150–170 *μ*m.

Minimal manual handling steps are required for cell culture using the Organ-Disc technology. Due to the small size of the reservoirs, in the current setting, fresh medium had to be refilled into the inner compartment and the outer reservoir, containing the effluent, emptied every day. However, using larger reservoirs, this interval could be prolonged significantly. As all compartments are accessible from the top, the reservoir can remain attached to the disc during medium exchange. In principle, this allows for the integration of liquid handling for medium exchange, eliminating all required manual handling during on-disc culture. Leveraging our user-friendly centrifugal perfusion process, ASCs were cultured in the Organ-Disc for up to three days maintaining viable 3D cell constructs [Fig. 3(e)]. This indicates that an even longer culture period on the Organ-Disc is in principle achievable. However, the suitability of our centrifugal perfusion for long-term culture as well as maintaining physiologic gas concentrations in our thermoplastic system will require further exploration.

**FIG. 4.**
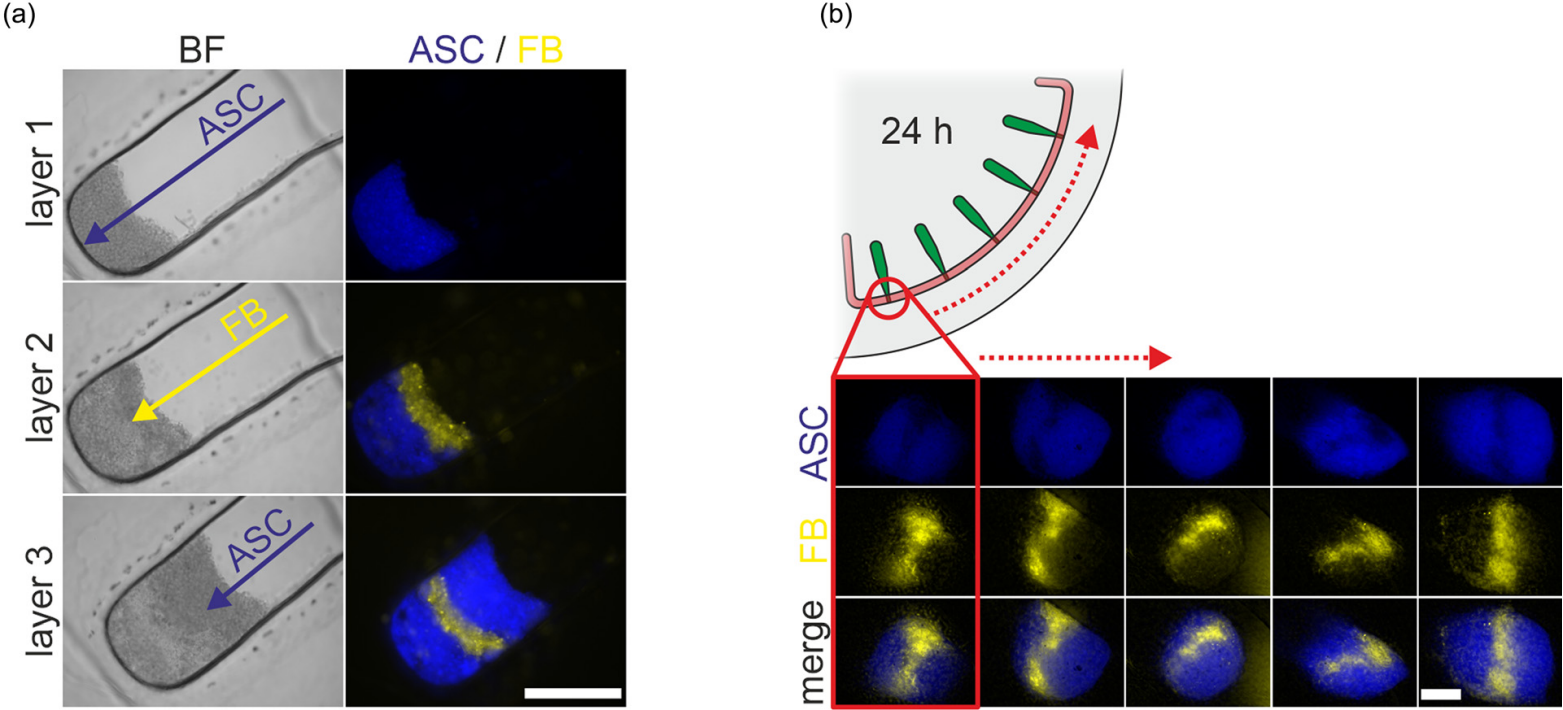
Multi-cell type construct generation: (a) bright field (BF) and fluorescence images of repeated cell loading of fluorescently labeled ASCs and FBs into a rectangular tissue chamber. (b) Fluorescence images of all the five chambers of one system of an Organ-Disc with stratified ASC/FB constructs cultured for 24 h (100 rpm, 0.46 g). The cell constructs maintained their targeted layer structure. Scale bars: 500 *μ*m.

In addition to only culturing one individual cell type at a time on the Organ-Disc, we used the Organ-Disc technology to generate and culture stratified constructs consisting of several cell types. Our rotation-based loading process enables sequential cell injection, as the loading is independent of the number of cells present in the tissue chamber and is, therefore, easily repeatable. Three batches of cells were loaded one after each other into each tissue chamber of the Organ-Disc. For each layer, 5000 cells were loaded into each chamber. Compared to our single-cell type loading, higher forces (approximately 100 g) were used for denser cell packing in order to achieve distinctly separated cell layers. The stepwise loading of ASCs and FBs resulted in a layered cell pellet. The different (fluorescently labeled) cell types were clearly distinguishable and separated in neighboring layers [Fig. 4(a)]. Even after 24 h of centrifugal culture, the layered cell constructs maintained their intended stratification with distinct layers of ASCs and FBs [Fig. 4(b)]. The generation of stratified, multi-cell type constructs paves the way for emulating organs and tissues that are built up from multiple layers with distinct properties and cell composition, such as retina, liver, or bone tissue.

## CONCLUSION

We have combined centrifugal microfluidics with OoC concepts, to establish the novel Organ-on-a-disc technology that allows for the parallelized generation and culture of human 3D cell constructs solely based on rotation. The Organ-Disc enables efficient cell loading, simultaneously generating 20 cell pellets with precisely controllable centrifugal forces. By eliminating the need for high pressure gradients or fluid flow during cell injection, cell damage is prevented and injection process efficiency increased. Using the Organ-Disc, we generated viable and dense 3D cell constructs, having a multi-cellular, tissue-like structure. Further, the injected cells adapt to a 3D shape, predefined by the tissue chamber geometry. We demonstrated the flexibility of our technology by introducing several cell types, compacting into a customizable geometry. In addition, we showed the usability of our system for targeted cell patterning, resulting in tailored 3D co-cultures. Thereby, we generated stratified, multi-cell type constructs using the Organ-Disc. This will enable emulating organ and tissue structures that are built up from layers of differing cell types or other specific properties like, e.g., stiffness. Going one-step further in the direction of automated microphysiological systems, we introduced pump and tubing-free centrifugal perfusion. We accomplished a centrifugal media supply just by slowly rotating the Organ-Discs. This enables a continuous and controllable vasculature-like perfusion of cell culture media to the cells. Our perfusion technique generates only minimal centrifugal forces (<1 g) acting on the cells. However, during perfusion, low but permanent centrifugal forces are required and inevitably. Nevertheless, our centrifugal perfusion approach allowed us to maintain viable cell constructs for up to three days and, in principle, is suitable for longer, culture periods. Furthermore, we developed the system based on industry-compatible thermoplastics and scalable, fabrication techniques. A thermoplastic chip will result in lower gas permeability and, hence, reduced oxygen concentrations in the tissue chambers compared to PDMS systems. However, the physiological relevance of oxygen levels in conventional cell culture and PDMS chips is controversially discussed since these levels are far above *in vivo* oxygen tensions. Additionally, accessibility of 3D cell constructs for off-chip analysis is more challenging in a thermoplastic housing. However, as the Organ-Disc fabrication can be scaled up, it will allow us to further explore the capabilities of the system, as, e.g., long-term culture experiments. Overall, the Organ-Disc provides a parallelizable and automatable platform technology for microphysiological systems, bringing OoCs closer to their intended revolution of next-generation drug development and personalized medicine.

## METHODS

### Organ-Disc and reservoir fabrication

**FIG. 5.**
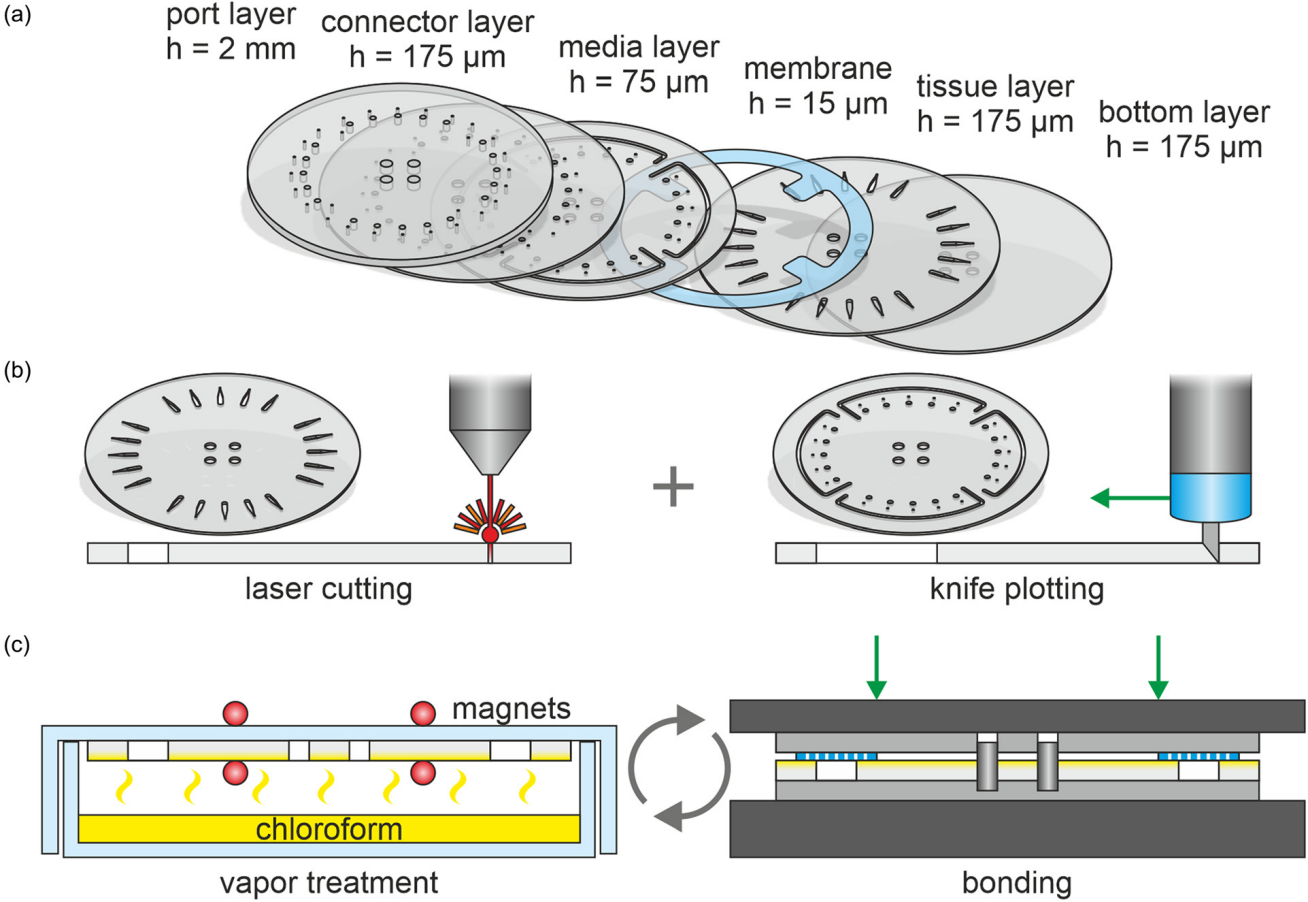
Organ-Disc fabrication process: (a) exploded view of the individual layers of the Organ-Disc. Each disc was built out of five microstructured PMMA layers and a porous PET membrane, separating media and tissue channels. (b) Utilized microfabrication techniques. Most layers were microstructured by CO_2_ laser cutting. Solely, the media layer was structured using a cutting plotter. (c) Process steps of the bonding procedure. The stepwise bonding consisted of a solvent vapor treatment using chloroform for generating a sticky surface and subsequent pressure bonding with a hydraulic press.

Organ-Discs were fabricated using five thermoplastic layers (polymethyl methacrylate; PMMA) and an isoporous membrane (polyethylene terephthalate; PET): the PMMA layers were a 2 mm thick port layer (Oroglas cast acrylic glass, Arkema), a 175 *μ*m thick connector, tissue and bottom layers (PLEXIGLAS Resist Clear 99524 GT, Röhm), and a 75 *μ*m thick media layer (PLEXIGLAS Film 0F072, Röhm). The 15 *μ*m thick, track-etched PET membrane with pores of 3 *μ*m in diameter (030444, SABEU) was sandwiched in between media and the tissue layer [Fig. 5(a)].

The PMMA foils and PET membrane were structured using a carbon dioxide (CO_2_) laser cutter (VLS2.30, Universal Laser Systems) or, in the case of the media layer, a cutting plotter (Graphtec CE6000–40 Plus, Graphtec) [Fig. 5(b)]. After structuring, cutouts were removed with tweezers and residues from the structuring processes were wiped away using cleanroom tissues soaked in isopropanol. Prior to bonding, the individual layers were blown with compressed nitrogen.

We used a stepwise fabrication approach, stacking microstructured layers gradually above each other. The stepwise process is based on an optimized solvent vapor treatment for surface roughness reduction and bonding.^44^ For the solvent exposure, a glass Petri dish (Anumbra, 120 × 20 mm^2^, neoLab) was filled with chloroform (pure, stabilized with ethanol, neoLab). In each consecutive bonding step, a PMMA layer was attached to the lid of the Petri dish with small magnets and placed over the chloroform-filled dish [Fig. 5(c)]. After 4 min of solvent vapor exposure, the PMMA surface became sticky. The exposed layer was then placed into a jig together with the respective counterpart and laminated on top of it using a handheld roller (Feed roller, STEINEL). The jig, used for facilitated alignment of individual layers, consisted of an 8 mm thick PMMA plate and pins cut from a 4 mm thick aluminum rod. After lamination, the jig was closed with another 8 mm thick PMMA plate and sandwiched in between two 2 mm thick silicone mats (Elastomer plate VMQ 50 Shore A, Angst+Pfister). The assembly was pressed together using a hydraulic press (LabEcon 150, Fontijne Presses) [Fig. 5(c)]. PMMA–PMMA connections were pressed with 50 kN and PMMA–PET bonds with 25 kN for 10 min each. We added a connector layer for better bonding strength in between the port and the media layer material out of the same material as tissue and bottom layers. Fully assembled and bonded Organ-Discs were left in a vacuum oven (Model VD 23, Binder) overnight at 60 °C and 20 mbar for solvent removal.

The Organ-Disc reservoir consists of three layers of PMMA: a 2 mm layer for bottom and lid and an 8 mm layer for the sidewalls (all Oroglas cast acrylic glass, Arkema). The reservoir compartments and openings in bottom and lid were again laser cut and bonded using glue (45570, UHU). Reservoirs were left overnight at room temperature for complete curing, then thoroughly rinsed with isopropanol, and dried under a laminar flow cabinet.

### Cell culture

Human, juvenile FBs from foreskin and ASCs from adult skin biopsies were utilized in this work. The biopsies were obtained from volunteers under informed consent according to the permission of the “Landesärztekammer Baden-Württemberg” (F-2012–078). All procedures were carried out in accordance with the rules for medical research of human subjects, as defined in the Declaration of Helsinki.

The cell isolation of FBs and ASCs was conducted as described previously.^45,46^ Both cell types were expanded in 75 cm^2^ filter cap cell culture flasks (CELLSTAR, Greiner Bio-One). FBs were cultured in Dulbecco's modified Eagle's medium (DMEM FG0445, Biochrom) supplemented with 10% (v/v) fetal bovine serum (FBS) (HyClone FetalClone II, Thermo Scientific) and 1% (v/v) penicillin/streptomycin (P/S) (10.000 U/ml, Gibco). ASCs were cultured in mesenchymal stem cell medium enhanced, serum-free (PB-C-MH-675–0511-ad, PELOBiotech), and 1% (v/v) antibiotic-antimycotic (100X, Thermo Fisher Scientific).

For passaging and cell suspension preparation, adherent cells were washed with phosphate buffered saline (PBS) (Dulbecco's phosphate buffered saline w/o calcium w/o magnesium, Biowest) and dissociated using 0.05% (v/v) trypsin (Trypsin-EDTA Solution 10X, SIGMA Life Science) in Versene solution (Versene 1:5000 1X, Gibco). After 2–3 min of trypsin incubation under culture conditions, the dissociation reaction was stopped by adding 10% FBS and the dissociated cells were transferred into a centrifuge tube (50 ml CELLSTAR Polypropylene Tube, Greiner Bio-One). The viable amount of cells was determined using trypan blue (Trypan blue 4 g/l in aqueous solution, VWR chemicals) and a hemocytometer (C-Chip Neubauer improved DHC-N01, NanoEnTek). After centrifugation (Multifuge 3S-R, Heraeus) at 1000 rpm (216 g), cells were resuspended to the final loading concentration in the respective cell culture medium.

### Organ-Disc spinner

For rotation of the Organ-Disc, we built two different setups: (i) a “loading spinner” for fast rotation, used for wetting of the channels and chambers, cell loading, as well as initialization of perfusion and (ii) a “perfusion spinner” for slow rotation during the continuous perfusion of the on-disc cell culture.

The core of the loading spinner is a brushless DC motor (QBL4208–41-04–006, TRINAMIC Motion Control) regulated by a speed controller (367 661, maxon motor) used for rotation speeds from 500 to 4000 rpm. The motor was integrated in a PMMA enclosure that fits under a standard laminar flow cabinet, allowing for an integration into sterile workflows (supplementary material Fig. 1).

The perfusion spinner integrates a stepper motor (SY42STH38–1684A, Pololu Corporation) controlled by a motor driver (2128, Pololu Corporation), used for rotation speeds of up to 200 rpm. The motor was fixed to a PMMA baseplate, which can be attached to an incubator shelve (Heraeus BBD 6220, Thermo Scientific), allowing for Organ-Disc spinning under culture conditions. All other electronic parts remained outside the incubator, connected by a thin cable to the motor. In both spinner setups, a LCD display (I2C 16 × 2 LCD Display Module, Eckstein), a rotary encoder (KY-040, reichelt elektronik), and a microcontroller (ATmega328P Board, Eckstein) provide a user interface for adjusting rotation parameters.

### Centrifugal cell loading

Prior to cell loading, Organ-Discs were sterilized and hydrophilized via a 5 min oxygen (O_2_) plasma treatment (<2 mbar, 3.3 sccm O_2_, 50 W) using a plasma generator (Zepto, Diener). Subsequently, all channels of the Organ-Disc were prefilled with PBS by filling media channels and all tissue channel inlets with a micropipette (10–100 *μ*l, VWR collection). If air remained entrapped in the dead-end tissue channels after initial filling by pipetting, those air bubbles could be easily removed by fast rotation of the Organ-Disc for 5 min at 4000 rpm (743 g at the tissue chambers). Thus, centrifugal forces transported entrapped air to the inlets of the tissue channel in the central disc region. For single-cell type loading, 5 *μ*l of cell suspension with 4000 cells/*μ*l were pipetted into the inlet region of each tissue channel. Subsequently, the Organ-Disc was rotated for 5 min at 1000 rpm (46.4 g). If the final amount of cells inside a tissue chamber was not sufficient for complete filling of a tissue chamber, the cell amount was adjusted by repeating the loading process a second time.

### Multi-cell type loading

To generate layered co-cultures in the Organ-Discs, we conducted three subsequent loading steps, in which first ASCs, then FBs, and again ASCs were introduced, by adopting a protocol from the study by Park *et al.*^29^ In each loading step, we used 5 *μ*l of cell suspension with 1000 cells/*μ*l and spun the Organ-Disc for 5 min at 1500 rpm (104.4 g). In between every loading step, the Organ-Disc was left in an incubator for 2 h for initial cell aggregation. Medium-filled pipette tips were attached to the medium ports, providing gravity-driven flow, in order to minimize evaporation and air bubble formation during this short incubation step. Different from the mono-culture experiments, for the co-culture, both ASCs and FBs were cultured using DMEM supplemented with 10% FBS and 1% P/S before and during on-disc co-culture.

Both cell types were stained using different fluorescence cell-labeling dyes before loading in order to distinguish between ASCs and FBs. Adherent ASCs were incubated with 12.5 *μ*M labeling solution (CellTracker Green CMFDA Dye, Thermo Fisher Scientific) in FBS-free DMEM for 45 min at culture conditions. Afterward, the labeling solution was aspirated and replaced with cell culture medium. FBs were labeled during passaging. After dissociation and resuspension (1 × 10^6^ cells/ml in FBS-free DMEM), we added 5 *μ*l of labeling solution (Vybrant DiD Cell-Labeling Solution, Thermo Fisher Scientific) per 1 ml of cell suspension and incubated for 20 min under culture conditions. To wash out the labeling solution, the cells were centrifuged (5 min 1500 rpm or 485 g), the supernatant was aspirated, and the cells were resuspended in medium twice. Patterned cell constructs on the Organ-Disc were imaged using a fluorescence microscope (BZ-X800, Keyence).

### Centrifugal media perfusion

Right after cell loading, all ports of the Organ-Disc were topped up with medium. The reservoir was attached to the Organ-Disc using double-sided adhesive tape (ARcare 90106, Adhesives Research) for a strong and permanent connection during the experiment runtime. Alternatively, we connected the reservoir reversibly by clamping a 2 mm thick layer of silicone (Sylgard 184, Dow Corning) in between the reservoir and the Organ-Disc. Silicone was mixed in a 10:1 (base: curing agent) mass ratio and cured for at least 4 h at 60 °C (Universal Oven UN110, Memmert). Finally, silicone was cut in the shape of the reservoir's bottom layer.

Prior to centrifugal media perfusion, the inner reservoir compartment was filled with medium and the Organ-Disc was spun for 30 s to flush the media channel with medium (500–1000 rpm or 11.6 g–46.4 g, respectively). Next, the Organ-Disc and reservoir were mounted on the perfusion spinner and continuous rotation under culture conditions was started. Each day, the centrifugal perfusion was stopped and the Organ-Disc was removed from the perfusion spinner. The effluent of each system was aspirated, and the cells were inspected using an inverted light microscope with a tempered enclosure at 37 °C (Leica DMi8, Leica Microsystems). Afterward, medium was refilled and centrifugal perfusion restarted.

### Flow rate measurements

In order to determine the flow rate generated by centrifugal pumping, we used a simplified version of the Organ-Disc containing only media channels. The procedure for plasma treatment, filling, and reservoir connection was identical to the handling of the standard Organ-Discs. The inner compartment of the reservoir was filled with de-ionized water that was preheated to 37 °C. Rotation speeds were varied from 0 rpm to 200 rpm, corresponding to centrifugal accelerations of 0 g–1.9 g. After 1 h of rotation under culture conditions, the flow rate was determined by weighing the effluents of each system using a fine balance (40SM-200A, Precisa Gravimetrics). OriginPro (Version 2019b, OriginLab Corporation) was used for error-weighted approximation of the measured flow rates.

### Live/dead staining

Cell viability after on-disc culture was evaluated using fluorescein diacetate (FDA, Sigma-Aldrich) and propidium iodide (PI, Sigma-Aldrich) staining. FDA (1 mg/ml in acetone) and PI (1 mg/ml in PBS) were diluted in PBS to a final concentration of 0.025 mg/ml FDA and 0.225 mg/ml PI. The Organ-Disc's medium channels were washed with PBS via gravity-driven flow and induced by pipette tips filled with PBS, which were attached to the media channels. Afterward, the staining solution was injected in the same way, incubated for 3 min at 37 °C, and finally removed by another PBS-wash. Z-stack images of stained 3D cell constructs were acquired at 37 °C using a fluorescence microscope with heated enclosure (Leica DMi8, Leica Microsystems). Maximum intensity projections were generated from the acquired z-stacks with Fiji (Image J version 1.52p–1.53c).^47^

### Actin/DNA staining

Cell constructs were fixed by flushing media channels with a fixative solution (Roti-Histofix 4%, Carl Roth). The fixative was incubated for 1 h at room temperature and removed by subsequent flushing with PBS. Cell structures were visualized by fluorescence staining of filamentous actin and nuclei using fluorescently labeled phalloidin (Alexa Fluor 488 phalloidin, Invitrogen) and 4′,6-Diamidino-2-phenylindole dihydrochloride (DAPI, Sigma-Aldrich), respectively. Fixated cell constructs were permeabilized for 30 min by injecting 0.1% (v/v) Triton X-100 in PBS (Triton X-100, Sigma-Aldrich) through the media channels and subsequent flushing with PBS. The staining solution was based on PBS supplemented with final concentrations of 0.165 *μ*M phalloidin, 1 *μ*g/ml DAPI, 1% (w/v) bovine serum albumin (BSA, Sigma-Aldrich), and 0.1% (v/v) Triton X-100. The staining solution was injected through the media channels, incubated for 60 min at room temperature and subsequently flushed out with PBS. The 3D cell constructs were imaged using a confocal Laser-Scanning-Microscope (LSM 710, Carl Zeiss MicroImaging). Combined z-stacks and tile scans were laterally stitched with ZEN (ZEN black edition 2.3 SP1, Carl Zeiss Microscopy). Subsequently, the stitched z-stacks were transformed into maximum intensity projections in vertical and lateral directions with Fiji.

## SUPPLEMENTARY MATERIAL

See the supplementary material for a figure showing the loading spinner for wetting, cell loading as well as initialization of perfusion of the Organ-Disc.

## Data Availability

The data that support the findings of this study are available from the corresponding author upon reasonable request.

## References

[1] B. Zhang , A. Korolj , B. F. L. Lai , and M. Radisic , Nat. Rev. Mater. 3, 257 (2018). 10.1038/s41578-018-0034-7

[2] S. Reardon , Nature 523, 266 (2015). 10.1038/523266a 26178942

[3] S. N. Bhatia and D. E. Ingber , Nat. Biotechnol. 32, 760 (2014). 10.1038/nbt.2989 25093883

[4] K. H. Benam , R. Villenave , C. Lucchesi , A. Varone , C. Hubeau , H.-H. Lee , S. E. Alves , M. Salmon , T. C. Ferrante , J. C. Weaver , A. Bahinski , G. A. Hamilton , and D. E. Ingber , Nat. Methods 13, 151 (2016). 10.1038/nmeth.3697 26689262

[5] J. Rogal , C. Binder , E. Kromidas , J. Roosz , C. Probst , S. Schneider , K. Schenke-Layland , and P. Loskill , Sci. Rep. 10, 6666 (2020). 10.1038/s41598-020-63710-4 32313039 PMC7170869

[6] A. Mathur , P. Loskill , K. Shao , N. Huebsch , S. Hong , S. G. Marcus , N. Marks , M. Mandegar , B. R. Conklin , L. P. Lee , and K. E. Healy , Sci. Rep. 5, 8883 (2015). 10.1038/srep08883 25748532 PMC4352848

[7] A. van den Berg , C. L. Mummery , R. Passier , and A. D. van der Meer , Lab Chip 19, 198 (2019). 10.1039/C8LC00827B 30506070 PMC6336148

[8] L. M. Mayr and D. Bojanic , Curr. Opin. Pharmacol. 9, 580 (2009). 10.1016/j.coph.2009.08.004 19775937

[9] C. Probst , S. Schneider , and P. Loskill , Curr. Opin. Biomed. Eng. 6, 33 (2018). 10.1016/j.cobme.2018.02.004

[10] M. W. Toepke and D. J. Beebe , Lab Chip 6, 1484 (2006). 10.1039/b612140c 17203151

[11] B. J. van Meer , H. de Vries , K. S. A. Firth , J. van Weerd , L. G. J. Tertoolen , H. B. J. Karperien , P. Jonkheijm , C. Denning , A. P. IJzerman , and C. L. Mummery , Biochem. Biophys. Res. Commun. 482, 323 (2017). 10.1016/j.bbrc.2016.11.062 27856254 PMC5240851

[12] D. T. T. Phan , X. Wang , B. M. Craver , A. Sobrino , D. Zhao , J. C. Chen , L. Y. N. Lee , S. C. George , A. P. Lee , and C. C. W. Hughes , Lab Chip 17, 511 (2017). 10.1039/C6LC01422D 28092382 PMC6995340

[13] N. R. Wevers , R. van Vught , K. J. Wilschut , A. Nicolas , C. Chiang , H. L. Lanz , S. J. Trietsch , J. Joore , and P. Vulto , Sci. Rep. 6, 38856 (2016). 10.1038/srep38856 27934939 PMC5146966

[14] K. Domansky , W. Inman , J. Serdy , A. Dash , M. H. M. Lim , and L. G. Griffith , Lab Chip 10, 51 (2010). 10.1039/B913221J 20024050 PMC3972823

[15] I. Maschmeyer , A. K. Lorenz , K. Schimek , T. Hasenberg , A. P. Ramme , J. Hübner , M. Lindner , C. Drewell , S. Bauer , A. Thomas , N. S. Sambo , F. Sonntag , R. Lauster , and U. Marx , Lab Chip 15, 2688 (2015). 10.1039/C5LC00392J 25996126

[16] C. Lohasz , N. Rousset , K. Renggli , A. Hierlemann , and O. Frey , SLAS Technol. 24, 79 (2019). 10.1177/2472630318802582 30289726

[17] R. Novak , M. Ingram , S. Marquez , D. Das , A. Delahanty , A. Herland , B. M. Maoz , S. S. F. Jeanty , M. R. Somayaji , M. Burt , E. Calamari , A. Chalkiadaki , A. Cho , Y. Choe , D. B. Chou , M. Cronce , S. Dauth , T. Divic , J. Fernandez-Alcon , T. Ferrante , J. Ferrier , E. A. FitzGerald , R. Fleming , S. Jalili-Firoozinezhad , T. Grevesse , J. A. Goss , T. Hamkins-Indik , O. Henry , C. Hinojosa , T. Huffstater , K.-J. Jang , V. Kujala , L. Leng , R. Mannix , Y. Milton , J. Nawroth , B. A. Nestor , C. F. Ng , B. O'Connor , T.-E. Park , H. Sanchez , J. Sliz , A. Sontheimer-Phelps , B. Swenor , G. Thompson , G. J. Touloumes , Z. Tranchemontagne , N. Wen , M. Yadid , A. Bahinski , G. A. Hamilton , D. Levner , O. Levy , A. Przekwas , R. Prantil-Baun , K. K. Parker , and D. E. Ingber , Nat. Biomed. Eng. 4, 407 (2020). 10.1038/s41551-019-0497-x 31988459 PMC8011576

[18] V. Lecault , M. VanInsberghe , S. Sekulovic , D. J. H. F. Knapp , S. Wohrer , W. Bowden , F. Viel , T. McLaughlin , A. Jarandehei , M. Miller , D. Falconnet , A. K. White , D. G. Kent , M. R. Copley , F. Taghipour , C. J. Eaves , R. K. Humphries , J. M. Piret , and C. L. Hansen , Nat. Methods 8, 581 (2011). 10.1038/nmeth.1614 21602799

[19] R. Gómez-Sjöberg , A. A. Leyrat , D. M. Pirone , C. S. Chen , and S. R. Quake , Anal. Chem. 79, 8557 (2007). 10.1021/ac071311w 17953452

[20] K. Renggli and O. Frey , *Organ-on-a-Chip* ( Elsevier, 2020), pp. 393–427.

[21] P. Andersson , G. Jesson , G. Kylberg , G. Ekstrand , and G. Thorsén , Anal. Chem. 79, 4022 (2007). 10.1021/ac061692y 17472339

[22] N. Honda , U. Lindberg , P. Andersson , S. Hoffmann , and H. Takei , Clin. Chem. 51, 1955 (2005). 10.1373/clinchem.2005.053348 16081503

[23] S. O. Sundberg , C. T. Wittwer , C. Gao , and B. K. Gale , Anal. Chem. 82, 1546 (2010). 10.1021/ac902398c 20085301

[24] O. Strohmeier , M. Keller , F. Schwemmer , S. Zehnle , D. Mark , F. von Stetten , R. Zengerle , and N. Paust , Chem. Soc. Rev. 44, 6187 (2015). 10.1039/C4CS00371C 26035697

[25] R. Gorkin , J. Park , J. Siegrist , M. Amasia , B. S. Lee , J.-M. Park , J. Kim , H. Kim , M. Madou , and Y.-K. Cho , Lab Chip 10, 1758 (2010). 10.1039/b924109d 20512178

[26] S.-W. Lee , J. Y. Kang , I.-H. Lee , S.-S. Ryu , S.-M. Kwak , K.-S. Shin , C. Kim , H.-I. Jung , and T.-S. Kim , Sens. Actuators, A 143, 64 (2008). 10.1016/j.sna.2007.06.043

[27] I. Kubo , S. Furutani , and K. Matoba , J. Biosci. Bioeng. 112, 98 (2011). 10.1016/j.jbiosc.2011.03.016 21497547

[28] I. Kubo , S. Furutani , and H. Nagai , *ECS Transactions* ( ECS, Honolulu, HI, 2009), pp. 1–8.

[29] J. Park , G.-H. Lee , J. Yull Park , J. C. Lee , and H. C. Kim , Biofabrication 9, 045006 (2017). 10.1088/1758-5090/aa9472 29045238

[30] D. C. Duffy , H. L. Gillis , J. Lin , N. F. Sheppard , and G. J. Kellogg , Anal. Chem. 71, 4669 (1999). 10.1021/ac990682c

[31] N. Thomas , A. Ocklind , I. Blikstad , S. Griffiths , M. Kenrick , H. Derand , G. Ekstrand , C. Ellström , A. Larsson , and P. Andersson , in *Micro Total Analysis Systems 2000*, edited by van den Berg A., Olthuis W., and Bergveld P. ( Springer Netherlands, Dordrecht, 2000), pp. 249–252.

[32] N. Kim , C. M. Dempsey , J. V. Zoval , J.-Y. Sze , and M. J. Madou , Sens. Actuators, B 122, 511 (2007). 10.1016/j.snb.2006.06.026

[33] O. Schneider , L. Zeifang , S. Fuchs , C. Sailer , and P. Loskill , Tissue Eng. Part A 25, 786 (2019). 10.1089/ten.tea.2019.0002 30968738 PMC6535963

[34] N. Huebsch , P. Loskill , N. Deveshwar , C. I. Spencer , L. M. Judge , M. A. Mandegar , C. B. Fox , T. M. A. Mohamed , Z. Ma , A. Mathur , A. M. Sheehan , A. Truong , M. Saxton , J. Yoo , D. Srivastava , T. A. Desai , P.-L. So , K. E. Healy , and B. R. Conklin , Sci. Rep. 6, 24726 (2016). 10.1038/srep24726 27095412 PMC4837370

[35] P. Loskill , T. Sezhian , K. M. Tharp , F. T. Lee-Montiel , S. Jeeawoody , W. M. Reese , P.-J. H. Zushin , A. Stahl , and K. E. Healy , Lab Chip 17, 1645 (2017). 10.1039/C6LC01590E 28418430 PMC5688242

[36] E. Berthier , E. W. K. Young , and D. Beebe , Lab Chip 12, 1224 (2012). 10.1039/c2lc20982a 22318426

[37] H. Bruus , *Theoretical Microfluidics* ( Oxford University Press, Oxford, New York, 2008).

[38] K. Achberger , C. Probst , J. Haderspeck , S. Bolz , J. Rogal , J. Chuchuy , M. Nikolova , V. Cora , L. Antkowiak , W. Haq , N. Shen , K. Schenke-Layland , M. Ueffing , S. Liebau , and P. Loskill , ELife 8, e46188 (2019). 10.7554/eLife.46188 31451149 PMC6777939

[39] H. Cho , H.-Y. Kim , J. Y. Kang , and T. S. Kim , J. Colloid Interface Sci. 306, 379 (2007). 10.1016/j.jcis.2006.10.077 17141795

[40] Y. Ma , X. Cao , X. Feng , Y. Ma , and H. Zou , Polymer 48, 7455 (2007). 10.1016/j.polymer.2007.10.038

[41] M. Bauer , M. Ataei , M. Caicedo , K. Jackson , M. Madou , and L. Bousse , Microfluid. Nanofluid. 23, 86 (2019). 10.1007/s10404-019-2252-8

[42] P. J. Lee , P. J. Hung , and L. P. Lee , Biotechnol. Bioeng. 97, 1340 (2007). 10.1002/bit.21360 17286266

[43] J. R. Masters and G. N. Stacey , Nat. Protoc. 2, 2276 (2007). 10.1038/nprot.2007.319 17853884

[44] I. R. G. Ogilvie , V. J. Sieben , C. F. A. Floquet , R. Zmijan , M. C. Mowlem , and H. Morgan , J. Micromech. Microeng. 20, 065016 (2010). 10.1088/0960-1317/20/6/065016

[45] M. Pudlas , S. Koch , C. Bolwien , S. Thude , N. Jenne , T. Hirth , H. Walles , and K. Schenke-Layland , Tissue Eng. Part C 17, 1027 (2011). 10.1089/ten.tec.2011.0082 21774693

[46] A.-C. Volz , B. Huber , A. M. Schwandt , and P. J. Kluger , Differentiation 95, 21 (2017). 10.1016/j.diff.2017.01.002 28135608

[47] J. Schindelin , I. Arganda-Carreras , E. Frise , V. Kaynig , M. Longair , T. Pietzsch , S. Preibisch , C. Rueden , S. Saalfeld , B. Schmid , J.-Y. Tinevez , D. J. White , V. Hartenstein , K. Eliceiri , P. Tomancak , and A. Cardona , Nat. Methods 9, 676 (2012). 10.1038/nmeth.2019 22743772 PMC3855844

